# Partial Progesterone Deprivation Affects the Expression of Apoptosis-Specific Genes and Proteins in a Zone-Specific Manner in Rat Placenta

**DOI:** 10.7759/cureus.95988

**Published:** 2025-11-03

**Authors:** Mariam M Alawadhi, Aseel Al-farra, Narayana Kilarkaje, Maie D Al-Bader

**Affiliations:** 1 Department of Physiology, Health Sciences Center, Kuwait University, Jabriya, KWT; 2 Department of Anatomy, Health Sciences Center, Kuwait University, Jabriya, KWT

**Keywords:** basal zone, labyrinth zone, placenta, progesterone, apoptosis

## Abstract

Background

Progesterone maintains the well-being of both the placenta and the fetus. Low maternal progesterone levels are strongly correlated with smaller placentas and fetal growth restriction.

Aim

To investigate the possible effects of reduced progesterone levels-induced placental zone-specific apoptosis mechanisms, which may contribute to intrauterine growth retardation (IUGR).

Methods

Sprague-Dawley rats were divided into three groups (control, progesterone-reduced, and progesterone-restored groups). On gestation day (dg) 15, ovariectomy was performed in the progesterone-manipulated groups, and a subcutaneous mini pump was implanted to release estradiol (40 ng/h). Estradiol was also injected twice daily from 17-20 dg. The progesterone-reduced rats received approximately one-third of the average progesterone level daily from 15 dg to maintain the normal pregnancy. Maternal progesterone levels, fetal and placental weights, and the expression of placental pro- and anti-apoptotic markers (Tumor protein p53 (p53), Cyclin-dependent kinase inhibitor 1 (p21), p27Kip1 (p27), BCL2-associated X protein (Bax), B-cell lymphoma 2 (Bcl2), β-catenin, and cyclin D1) were measured on 16, 19, and 21 dg.

Key results

A 37% reduction in progesterone level was observed in the progesterone-reduced group on 19 dg. This decline was associated with a significant decrease in both whole placental and basal zone weights, while the fetal body weight and labyrinth zone weight remained unchanged. These changes occurred concurrently with increased placental expression of proapoptotic markers (p53, p21, p27, Bax) and antiapoptotic markers (cyclin D1 and Bcl2) in both placental basal and labyrinth zones at 16, 19, and 21 dg. Progesterone restoration recovered most of the molecular changes that emanated from its withdrawal.

Conclusion

The modulatory effect of progesterone did not result in improved fetal body weight, likely due to the variable impact of progesterone on the expression of pro- and anti-apoptotic proteins within the labyrinth and basal zones across different gestational days. Implication: These findings indicate that the effect of progesterone on cell death and survival pathways is zone-specific, reflecting different regulatory mechanisms within different placental regions.

## Introduction

Progesterone plays a crucial role in maintaining the well-being of both the placenta and the fetus during gestation. The abnormal decline in progesterone levels strongly correlates with intrauterine growth restriction (IUGR) in humans [1]. Animal models of IUGR also demonstrated a significant reduction in maternal progesterone levels [2,3]. IUGR is a pathological condition in which a fetus fails to reach its expected growth rate, leading to short- and long-term complications after birth.

Apoptosis is a normal process during placenta formation, starting from early pregnancy, and it increases with the progression of gestation. IUGR animal models demonstrated an upregulation in the expression of pro-apoptotic proteins Metastasis-Associated Protein 3 (MTA3), Tumor protein p53 (p53), and Cyclin-dependent kinase inhibitor 1 (p21) in both the labyrinth and basal zones. In contrast, the expression of the anti-apoptotic protein β-catenin was markedly downregulated, specifically in the labyrinth zone only [4,5]. The Tp53 gene plays a crucial role in maintaining genomic stability and regulates cell cycle progression by activating genes involved in DNA repair, growth arrest, and apoptosis. The p53 protein, through its ability to modulate p21 at the transcriptional level, induces cell cycle arrest, while p27Kip1 (p27), which is homologous to p21, has a role in inducing apoptosis and inhibiting cellular proliferation (reviewed in [6]).

The protein β-catenin is a component of the Wingless-related integration site (Wnt) signaling pathway. The Wnt/β-catenin pathway has a significant role in apoptosis, development, cellular proliferation, and differentiation. β-catenin also induces the expression of cyclin D1 [7], which controls cell cycle progression, inducing proliferation and cell migration in a variety of cells [8,9]. Cyclin D1 is also known to induce cell cycle arrest when induced by p53 and p21 [10]. The crosstalk between β-catenin and p53 appears to be important during tumorigenesis and DNA damage, during which process deregulation of β-catenin activates p53 [11]. Induction of p53 correlates with overexpression of cyclin D1 in carcinoma cells [12] and downregulation of cyclin D1 in other cells [13]. Both p21 and p27 are necessary for the nuclear translocation of cyclin D1, which indicates that cell cycle regulators are functionally linked.

Progesterone has a dual role in inducing proliferation and apoptosis in reproductive organs. The pro-proliferative function of progesterone is achieved by initiating Wnt/β-catenin signaling and inducing the expression of cyclin D1 and B-cell lymphoma 2 (Bcl2) in luteal and uterine cells [14,15]. Cell culture studies also show that progesterone increases the anti-apoptotic protein Bcl2 expression in different reproductive organ cells [16,17], thus protecting against apoptosis [18]. Progesterone withdrawal studies have also confirmed progesterone's anti-apoptotic role during the decidualization period of pregnancy [19]. Progesterone increases placental expression of pro-proliferative protein metastasis-tumor antigen-1 and reduces placental expression of pro-apoptotic protein metastasis-tumor antigen-3 in dexamethasone-induced IUGR rats [5]. Cell proliferative and apoptotic effects of progesterone are variable and most likely tissue- and dose-dependent [16,19] and is greatly dependent on other sex steroids, most importantly, estrogen [14].

The above studies show that progesterone withdrawal is related to decreased cell proliferation, leading to smaller placentas and IUGR. However, these studies addressed the role of progesterone withdrawal during early pregnancy only, not at the critical time of both placental and fetal growth during the third trimester (gestation day 19 or 19 dg when the placenta reaches its maximum growth and 21 dg when the fetus reaches its maximum intrauterine growth) [20]. In addition, previous studies that evaluated the role of apoptosis in IUGR were conducted at the end of gestation before labor. However, the role of progesterone in modulating the major pro-and anti-apoptotic proteins at an earlier age is unknown. Therefore, in the present study, we investigated the in vivo effect of progesterone on placental anti- and pro-apoptotic markers at three critical time points of placental and fetal development (16, 19, and 21 dg) using a rodent model of progesterone reduction and progesterone restoration [2].

## Materials and methods

Induction of progesterone deprivation in pregnant rats

The experimental design was modified from Mark et al. [2]. Based on previous studies [21,22], Sprague-Dawley rats (45 rats; 10-12 weeks old) were procured from the Animal Resources Center at the Health Sciences Center, Kuwait University. The rats had free access to food and water and were maintained in a standard environment (12:12 day/night cycle, 22-23°C, 40-50% humidity). The Health Sciences Research Ethics Committee approved this study in 2008. The rats were handled according to the National Institute of Health (NIH) and Animal Research: Reporting In Vivo Experiment (ARRIVE) guidelines during the experimental procedure. After overnight mating, the presence of sperm in the vaginal smear indicated pregnancy and was designated as 0 dg. Since the aim of this study was to evaluate the effect of progesterone on placental apoptosis, the experiment included a progesterone-reduced group, in which the progesterone level was reduced, and a progesterone-restored group, in which the progesterone level was maintained at a normal level during pregnancy. On 15 dg, the rats were randomly allocated to their groups, five per group for each time point. The groups were: a control group, a progesterone-reduced group, and a progesterone-restored group.

On 15 dg, ovariectomy was performed in the pregnant rats belonging to the progesterone-manipulated groups. The rats were anesthetized by administering a mixture of Ketamine (70 mg/kg) and Rompun (10 mg/kg; 2 µl/g body weight). A ventral midline incision was made on the abdominal wall, and the ovaries were identified and removed. The number of fetuses in each uterine horn was recorded. The pregnant rats with fetuses ranging from six to 13 were included in this study; rats with fetuses out of this range were excluded from the study, as the number of conceptuses below or higher than this range has a significant inverse relationship with the fetal weight [20]. Estradiol was replaced via a subcutaneous mini-osmotic pump that was implanted during the ovariectomy procedure. The pump rate was 40 ng/h in propylene glycol (Model 1006, Alzet, Sydney, Australia). This was followed by injections to mimic the average increase in plasma estradiol levels. The rats were placed on heating pads to maintain their body temperature during the procedure. Progesterone was administered daily to restore its circulating levels to the normal range (progesterone restoration) or reduced to approximately one-third of the normal range measured on 22 dg (progesterone reduction). Control group rats were sham-operated and received twice daily injections of vehicle (peanut oil). Details of the experimental injection procedure are provided in Table 1.

**Table 1:** Experimental design Details of the rat groups and the treatment protocol; dg: gestation day; s.c.: subcutaneous.

Progesterone treatment	15 dg	16 dg	17 dg	18 dg	19 dg	20 dg	21 dg
Control	(40 ng/hr) 1x daily s.c. of 0.2 ml peanut oil	2x daily s.c. of 0.2 ml peanut oil	2x daily s.c. of 0.2 ml peanut oil	2x daily s.c. of 0.2 ml peanut oil	2x daily s.c. of 0.2 ml peanut oil	2x daily s.c. of 0.2 ml peanut oil	
Progesterone-reduced group	Ovariectomy and implantation of subcutaneous (s.c.) mini pump with estradiol (40 ng/hr); 1x daily s.c. injections of progesterone (0.5 mg) in 0.2 ml peanut oil	2x daily s.c. injections of progesterone (0.5 mg) in 0.2 ml peanut oil	2x daily s.c. injections of estradiol (250 ng) in 0.2 ml peanut oil; 2x daily s.c. injections of progesterone (0.5 mg) in 0.2 ml peanut oil	2x daily s.c. injections of estradiol (250 ng) in 0.2 ml peanut oil; 2x daily s.c. injections of progesterone (0.5 mg) in 0.2 ml peanut oil	2x daily s.c. injections of estradiol (500 ng) in 0.2 ml peanut oil; 2x daily s.c. injections of progesterone (0.5 mg) in 0.2 ml peanut oil	2x daily s.c. injections of estradiol (500 ng) in 0.2 ml peanut oil; 2x daily s.c. injections of progesterone (0.5 mg) in 0.2 ml peanut oil	
Progesterone-restored group	Ovariectomy and implantation of subcutaneous (s.c.) mini pump with estradiol (40 ng/hr); 1x daily s.c. injections of progesterone (10 mg) 0.2 ml peanut oil	2x daily s.c. injections of progesterone (10 mg) in 0.2 ml peanut oil	2x daily s.c. injections of estradiol (250 ng) in 0.2 ml peanut oil; 2x daily s.c. injections of progesterone (10 mg) in 0.2 ml peanut oil	2x daily s.c. injections of estradiol (250 ng) in 0.2 ml peanut oil; 2x daily s.c. injections of progesterone (7.5 mg) in 0.2 ml peanut oil	2x daily s.c. injections of estradiol (500 ng) in 0.2 ml peanut oil; 2x daily s.c. injections of progesterone (5 mg) in 0.2 ml peanut oil	2x daily s.c. injections of estradiol (500 ng) in 0.2 ml peanut oil; 2x daily s.c. injections of progesterone (5 mg) in 0.2 ml peanut oil	
Experiment termination		Sample collection			Sample collection		Sample collection

Tissue collection

The experiment was terminated on 16, 19 (the time of maximum placental growth), and 21 (the time of maximum fetal growth) dg. The rats were anesthetized, as mentioned above, and a thoracotomy was conducted, and maternal blood was collected from the left ventricle. The thoracotomy incision was extended to the abdomen and then to the pelvis to expose the pelvic organs. Uterine horns containing conceptuses were removed and immediately placed on ice. Fetuses and placentas were separated on ice (five pregnancies per group were obtained at each gestational age). Placental labyrinth and basal zones have distinct structures and boundaries and were separated using forceps. The number of pups was counted, and the fetal body weight, the weights of the whole placenta, and the weights of the labyrinth and basal zones were taken. All placental zones from each dam were pooled and stored separately for further analysis. For protein analysis, the cryoprotective agent, dimethylsulfoxide (10% v/v), was added to the placental samples before freezing at -70°C.

Measurement of the maternal progesterone level

The maternal progesterone level was measured using a kit (CSB-E07282r, Cusabio Biotech Co., Ltd.) with a sensitivity of 0.2 ng/mL. The assay was performed according to the instructions given in the manual. Briefly, 50 µl of sample or standard was added to wells in a 96-well microplate. Then, 50 µl of Horseradish peroxidase (HRP)-conjugated progesterone and 50 µl of antibody were added to all the wells, except for the blank, and incubated for one hour at 37°C. The liquid was removed, and the wells were washed. The detector was added and incubated for 15 min at 37°C. The stop solution was added to each well, and the absorbance was read at 450 nm within 10 min. The data were fitted into a four-parameter logistics curve using the CurveExpert 1.3 software (Hymas Development, Tennessee, US).

Real-time polymerase chain reaction (ReT-PCR)

ReT-PCR was conducted to estimate gene expression according to the procedure described in our previous study [3]. RNA was extracted by the TRIzol method (TRIzol 15569-018, Invitrogen ThermoFisher Scientific, United States). The integrity and purity of samples were measured using agarose gel electrophoresis and Epoch Microplate Spectrophotometry-Epoch Tak 3 plate (BioTek Instruments, Vermont, US), respectively. Only integrated and pure samples (A260/A280 >1.7) were used for further analysis. The samples were DNase-treated and reverse-transcribed. Samples were run in an ReT-PCR system (Applied Biosystems, model 7500, Waltham, Massachusetts, US) for gene expression of Tp53 (Rn00755717), CDKN1A (p21 gene; Rn00589996), CDKN1B (p27 gene; Rn00852195), CCND1 (Cyclin D1 gene; Rn00432359), CTNNB1 (β-Catenin gene; Rn00584431), BCL2-associated X protein (Bax) (Rn01480161), and Bcl2 (Rn07313625_m1). Since the validity and stability of 18S (Rn01747392_m1) was proven previously [23], it was used as a housekeeping gene (Table 2).

**Table 2:** Primers and their specifications Primers sourced from Applied Biosystems (Waltham, Massachusetts, US).

Primer	Catalog no.	Primer sequence
Tp53	Rn00755717	Forward sequence: CCTCAGCATCTTATCCGAGTGG Reverse sequence: TGGATGGTGGTACAGTCAGAGC
CDKN1A	Rn00589996	Forward sequence: AGGTGGACCTGGAGACTCTCAG Reverse sequence: TCCTCTTGGAGAAGATCAGCCG
CDKN1B	Rn00852195	Forward sequence: ATAAGGAAGCGACCTGCAACCG Reverse sequence: TTCTTGGGCGTCTGCTCCACAG
BAX	Rn01480161	Forward sequence: TCAGGATGCGTCCACCAAGAAG Reverse sequence: TGTGTCCACGGCGGCAATCATC
BCL2	Rn07313625_m1	Forward sequence: CCT GTG GAT GAC TGA GTA CC Reverse sequence: GAG ACA GCC AGG AGA AAT CA
CTNNB1	Rn00584431	Forward sequence: CACAAGCAGAGTGCTGAAGGTG Reverse sequence: GATTCCTGAGAGTCCAAAGACAG
CCND1	Rn00432359	Forward sequence: TCTACACCGACAACTCCATCCG Reverse sequence: TCTGGCATTTTGGAGAGGAAGTG
18S	Rn01747392_m1	Forward sequence: CATTCGAACGTCTGCCCTAT Reverse sequence: GTTTCTCAGGCTCCCTCTCC

All reagents used in this study were purchased from Invitrogen Incorporated Inc. and Applied Biosystems (Waltham, Massachusetts, US), unless stated otherwise.

Western blotting

Western blotting and immunodetection were conducted to semi-quantify p53, β-catenin, cyclin D1, Bax, and Bcl2, as described previously [3]. Placental zones were washed and homogenized, the subcellular protein fraction was separated by ultracentrifugation as described previously [24], and the protein content was determined using Epoch microplate spectrophotometry. As most protein level changes were observed in the nuclear fraction, we focused on the nuclear expression of the cell cycle proteins. The molecular weight rainbow markers, a positive control sample, and samples (20 µg protein of nuclear fraction) were electrophoresed (SDS PAGE; 5-14% polyacrylamide gradient gels, Bio-Rad Laboratories, California, USA).

Proteins were transferred to polyvinylidene fluoride (PVDF) membranes (GE Healthcare Lifesciences, UK) and blocked for one hour at room temperature with 10% non-fat dry milk in TBS-T (20 mM Tris, 137 mM NaCl, pH 7.6, 0.1% v/v Tween 20), then incubated overnight at 4°C with the primary antibodies. The antibody specifications are given in Table 3.

**Table 3:** Primary and secondary antibody specifications and dilutions

Primary Antibody	Company	Catalog no.	Molecular Weight (kDa)	Primary Concentration	Secondary Antibody	Catalogue no.	Secondary Concentration
p53 Mouse Monoclonal	Invitrogen	MA5-12453	53	1:5000	Anti-Mouse IgG	A2554	1:10000
Bax Rabbit Monoclonal	Abcam	ab32503	21	1:3000	Anti-Rabbit IgG	Ab6721	1:10000
Bcl2 Rabbit Monoclonal	Abcam	ab32124	26	1:1000	Anti-Rabbit IgG	Ab6721	1:5000
β-catenin Rabbit Monoclonal	Abcam	ab32572	92	1:1000	Anti-Rabbit IgG	Ab6721	1:10000
Cyclin D1 Rabbit Monoclonal	Abcam	ab16663	33	1:1000	Anti-Rabbit IgG	Ab6721	1:5000
β-actin (C4) Mouse Monoclonal	MilliporeSigma	MAB1501R	42	1:10000	Anti-mouse IgG	A2554	1:20000

After washing, the membranes were incubated with the appropriate secondary antibodies for 1.5 h. Immunodetection was done by chemiluminescence using a kit (ECL-Plus, Amersham Pharmacia Biotech Ltd., UK). The density of the protein bands was estimated using the Bio-RAD ChemiDOCTM MP Imaging system (Bio-RAD, California, US). After obtaining the results, the membranes were re-probed for the loading control protein β-actin, as it proved its stability and use for the nuclear fraction [25]. All protein expressions were expressed relative to β-actin expression.

Statistical analysis

The relative gene expression was calculated according to the Livak method and as described in our previous study using the cycle threshold (Ct) data [26]. Briefly, the Ct value of the housekeeping gene (18S) was subtracted from that of the target gene to obtain the ΔCt. The ΔΔCt was calculated by subtracting the ΔCt of the calibrator group (16 dg control) from the ΔCt of each experimental sample. The normalized expression of the target gene was then determined using the formula 2-ΔΔCt. The protein expression was calculated as explained in our earlier study [26]. The optical density of a sample was divided by the optical density of β-actin. The IBM SPSS Statistics for Windows, Version 26 (Released 2019; IBM Corp., Armonk, New York, US) was used for statistical analysis, and the images were created using ImageJ software [27]. The homogeneity of variance was tested, and the data were analyzed by two-way ANOVA and the least-squares difference test (LSD). The Mann-Kendall test was used to evaluate the correlation and test trend change whenever necessary. Data were presented as mean ± S.E.M. for each group (n=5). The intergroup differences were considered statistically significant at a p<0.05.

## Results

Low progesterone level reduces placental basal zone weight but does not cause growth retardation in rats

**Figure 1:**
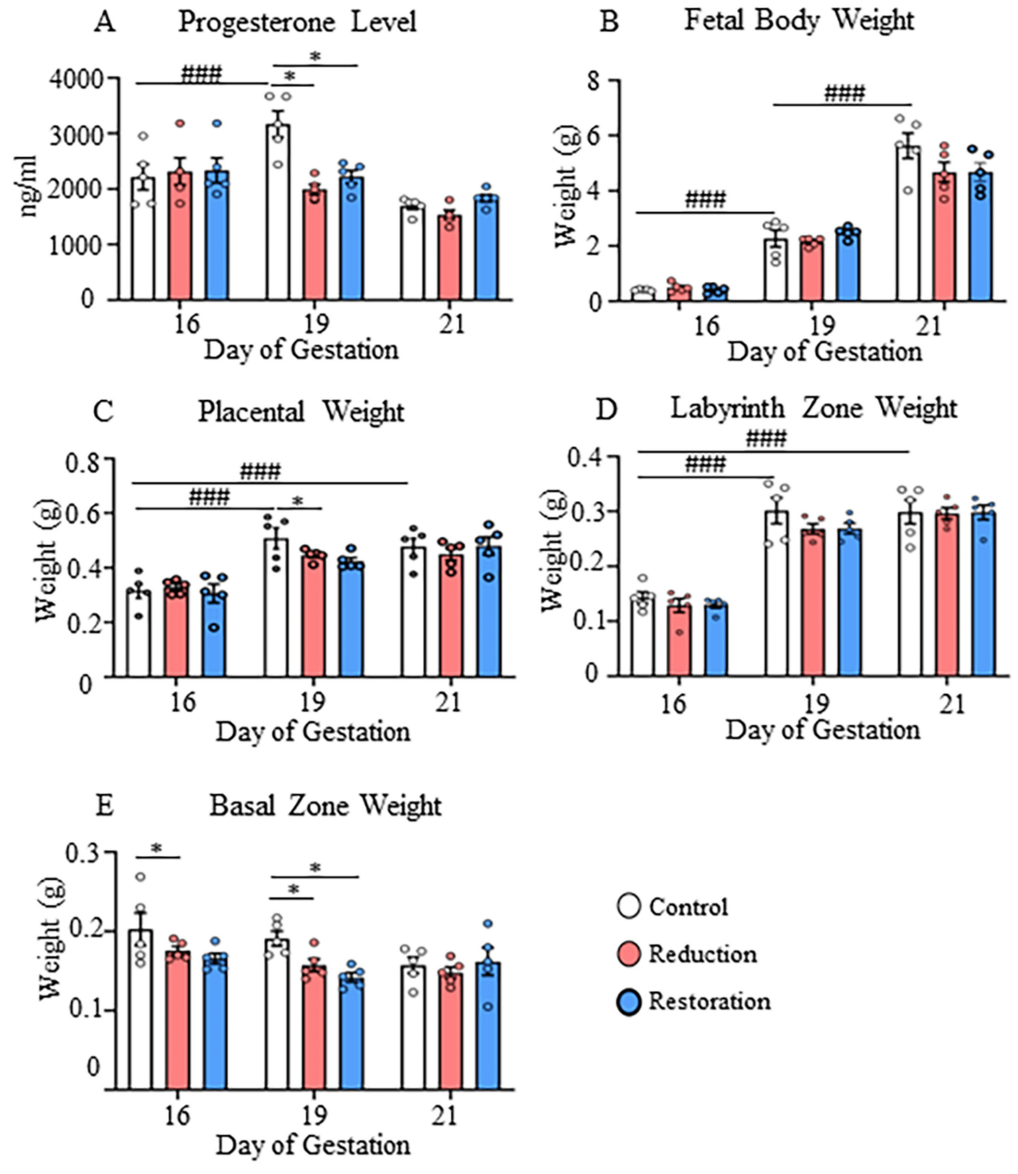
Gross parameters A) Maternal progesterone level, B) fetal body weight, C) placental weight, D) labyrinth zone weight, E) basal zone weight, and F) placental efficiency at 16, 19, and 21 dg in control, progesterone-reduced and restored-progesterone groups. ^*^Represents significant differences between groups at the same gestational age; ^#^represents significant differences between similar groups at different gestational ages using two-way ANOVA (^*^p<0.05; ^###^p<0.001). Data are represented as mean ± SEM (n=5). Kendall-Tau test was used to analyze the decreasing trend in fetal body weight by correlating the control and progesterone-reduced groups (the correlation was 0.8 and p=0.5)

The maternal progesterone level increased with the progression of gestation from 16 to 19 dg (p<0.05), then decreased by 21 dg in the control group (p<0.001; Figure 1A, Appendix A).

On 19 dg, the progesterone-reduced group had a significantly lower maternal progesterone level than the control group (p<0.01; Figure 1A). Progesterone supplementation did not affect the litter number or the uterine weight (data not shown). The fetal body weight increased significantly with the progression of gestation in all experimental groups (p<0.001; Figure 1B, Appendix A). The reduction in fetal body weight in the progesterone-reduced group at 21 dg did not reach statistical significance (p=0.07) but showed a trend to reduce (Mann-Kendall test; correlation=0.8, p=0.05). The placental and labyrinth zone weights increased from 16 to 19 dg and 21 dg in all treatment groups (p<0.001; Figures 1C, 1D, Appendix A), but without significant differences between 19 dg and 21 dg. In the progesterone-reduced group, the placental weight was reduced by 18% compared to the control group at 19 dg (p<0.05; Figure 1C). The basal zone weight was also significantly reduced in the progesterone-reduced group at 16 dg (18%) and 19 dg (22%) compared to the control group (p<0.05; Figure 1E). The labyrinth zone showed no progesterone level-dependent weight change at any gestational age (Figure 1D).

Progesterone deprivation increases the expression of pro-apoptotic and anti-apoptotic proteins in the placenta

**Figure 2:**
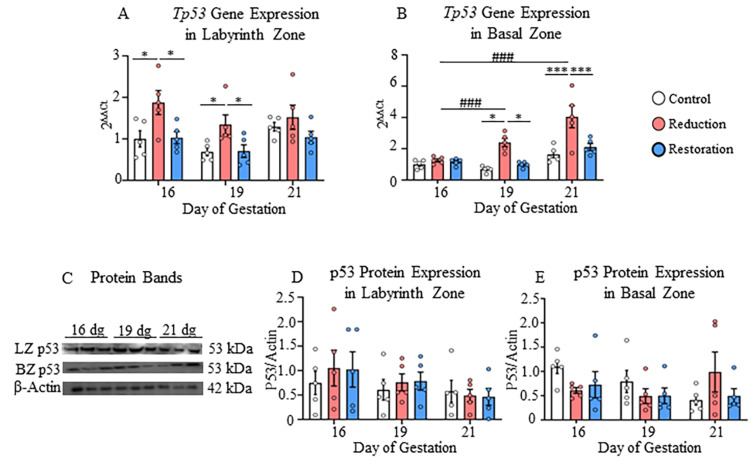
Gene and protein expressions of p53 in labyrinth and basal zones A) Gene expression of Tp53 in labyrinth and B) Basal zones; C) Protein bands of p53 and β-actin in the labyrinth and basal zones; D) Protein expression of p53 in labyrinth and E) Basal zones at 16, 19, and 21 dg in control, progesterone-reduced, and restored-progesterone groups. ^*^Represents significant differences between groups at the same gestational age; ^#^represents significant differences between similar groups at different gestational ages using two-way ANOVA (^*^p<0.05, ^***###^p<0.001). Data are represented as mean ± SEM (n=5)

The gene Tp53 and its downstream genes CDKN1A and CDKN1B were upregulated compared to the control in the progesterone-reduced and progesterone-restored groups at different gestational ages in both the labyrinth and basal zones. In the labyrinth zone, the Tp53 expression was significantly higher in the progesterone-reduced group than in other groups at 16 dg and 19 dg (p<0.05; Figure 2A).

**Figure 3:**
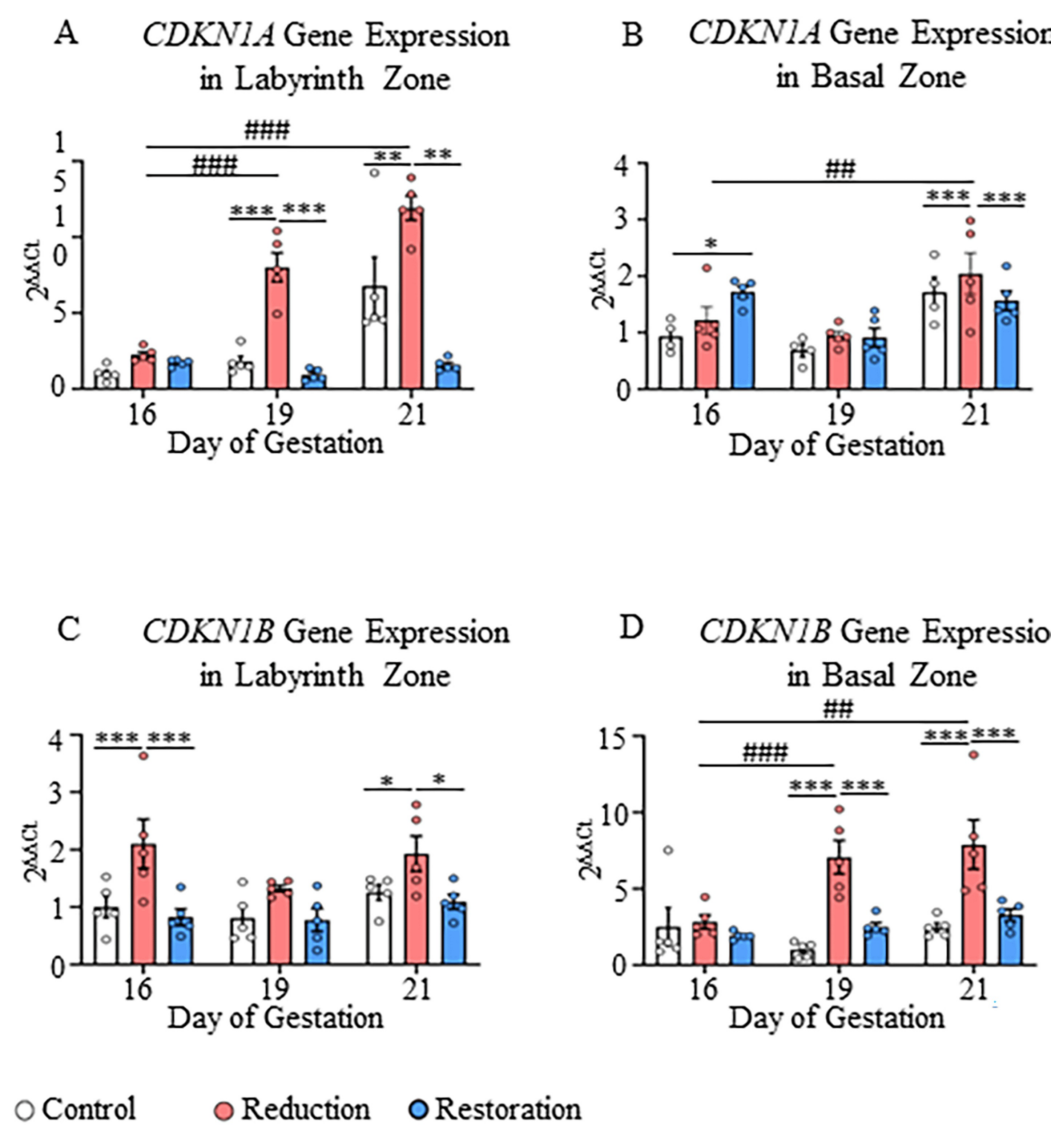
Gene expression of CDKN1A and CDKN1B in labyrinth and basal zones A) Gene expression of CDKN1A in labyrinth and B) Basal zones; C) Gene expression of CDKN1B in labyrinth and D) Basal zones at 16, 19, and 21 dg in control, progesterone-reduced, and restored-progesterone groups. ^*^Represents significant differences between groups at the same gestational age; ^#^represents significant differences between similar groups at different gestational ages using two-way ANOVA (^*^p<0.05, ^**##^p<0.01, ^***###^p<0.001). Data are represented as mean ± SEM (n=5).

Similarly, in the labyrinth zone, the gene expression was significantly higher in the progesterone-reduced group for both CDKN1A (at 19 dg and 21 dg, p<0.01-p<0.001; Figure 3A) and CDKN1B (at 16 dg and 21 dg; p<0.05-p<0.001; Figure 3D).

Progesterone level restoration decreased the expression of Tp53, and its downstream genes returned to normal expression levels. Both p21 and p27 proteins were not at detectable levels in both placental zones. In the basal zone, the Tp53 expression was increased towards the end of pregnancy (at 19 dg and 21 dg) in the progesterone-reduced group compared to the other groups (p<0.05-p<0.001; Figure 2B). The increase in Tp53 gene expression was also significant with the progression of gestation from 16 dg to 19 dg and 16 dg to 21 dg in the progesterone-reduced group in the basal zone (p<0.001; Figure 2B, Appendix B). The same finding was observed in the basal zone for CDKN1A and CDKN1B gene expression. Both genes showed an increased expression in the progesterone-reduced group compared to the control group towards the end of pregnancy (p<0.001; Figures 3B, 3E).

**Figure 4:**
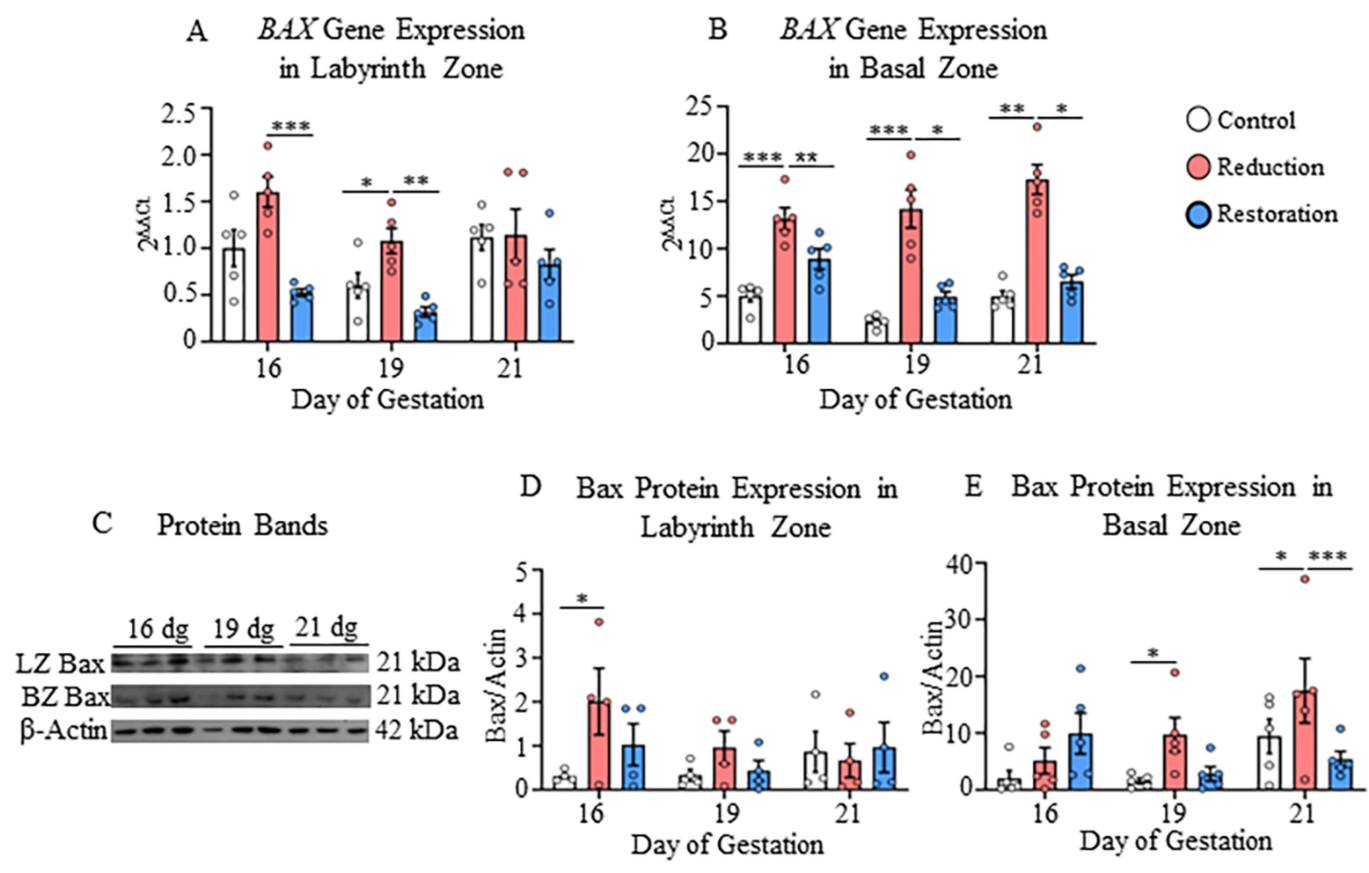
Gene and protein expressions of Bax in the labyrinth and basal zones A) Gene expression of Bax in the labyrinth and B) Basal zones; C) Protein bands of Bax and β-actin in the labyrinth and basal zones; D) Protein expression of Bax in labyrinth and E) Basal zones at 16, 19, and 21 dg in control, progesterone-reduced, and restored-progesterone groups. ^*^Represents significant differences between groups at the same gestational age using two-way ANOVA (^*^p<0.05, ^**^p<0.01, ^***^p<0.001). Data are represented as mean ± SEM (n=5)

The expression of Bax gene significantly increased in the labyrinth zone in the progesterone-reduced group at 16 dg and 19 dg (p<0.05-p<0.001; Figure 4A) and in the basal zone at 19 dg and 21 dg (p<0.01-p<0.001; Figure 4B) compared to the control group.

The gene expression in both basal and labyrinth zones was restored in progesterone-restored groups (p<0.05-p<0.01, Figures 4A, 4B). At the protein level, the changes were detected in both zones with significant upregulation of Bax at 16 dg (labyrinth zone) and 19 dg (basal zone) in the progesterone-reduced group compared to the control group (p<0.05; Figures 4D, 4E).

**Figure 5:**
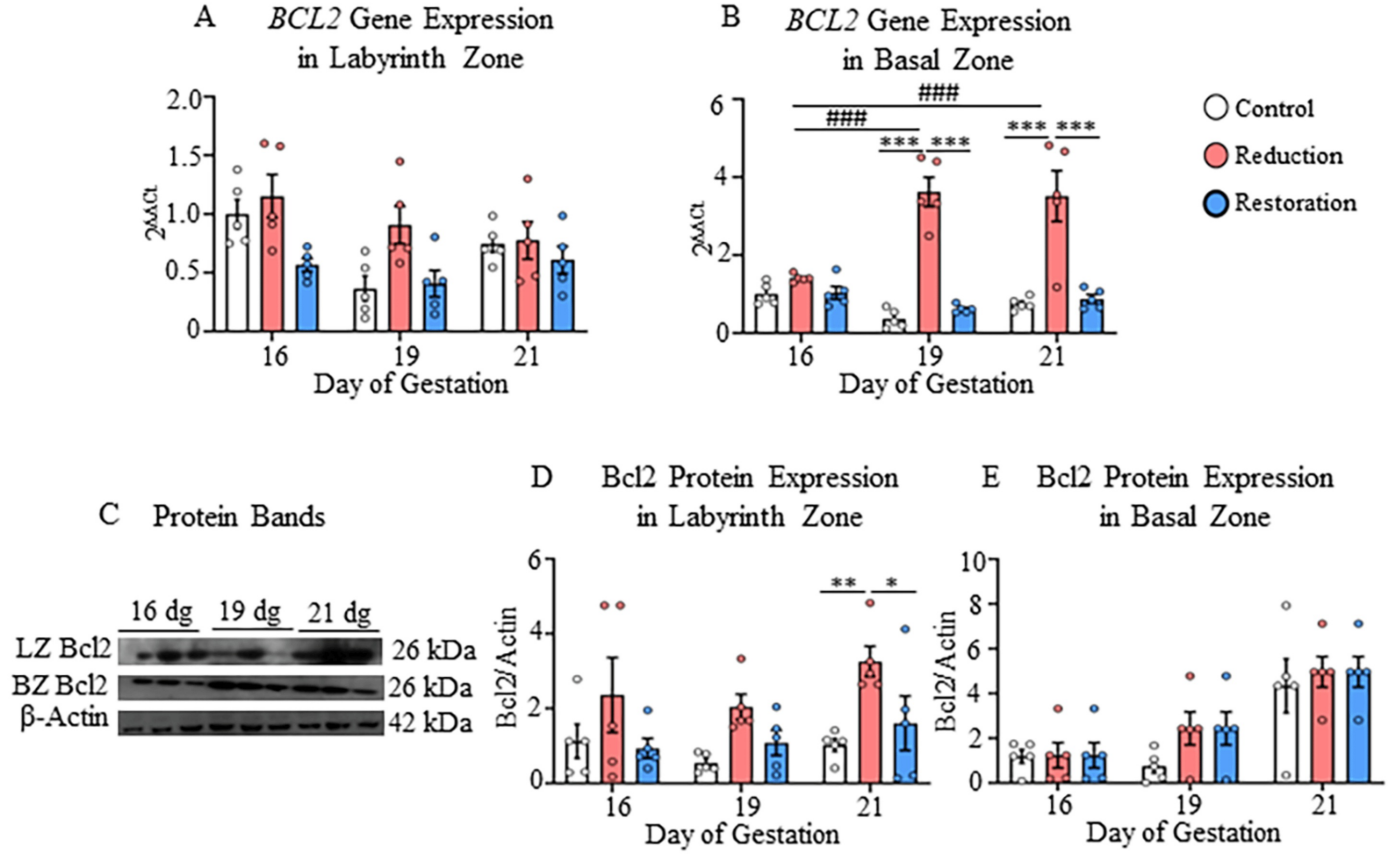
Gene and protein expressions of Bcl2 in labyrinth and basal zones A) Gene expression of Bcl2 in labyrinth and B) Basal zones; C) Protein bands of Bcl2 and β-actin in the nuclear fraction of labyrinth and basal zones; D) Protein expression of Bcl2 in labyrinth and E) Basal zones at 16, 19, and 21 dg in control, progesterone-reduced, and restored-progesterone groups. ^*^Represents significant differences between groups at the same gestational age; ^#^represents significant differences between similar groups at different gestational ages using two-way ANOVA (^*^p<0.05, ^**^p<0.01, ^***###^p<0.001). Data are represented as mean ± SEM (n=5)

The expression of anti-apoptotic marker Bcl2 was significantly higher in both placental zones in the progesterone-reduced group compared to other groups. The increased Bcl2 protein expression was seen at 21 dg in the labyrinth zone compared to both control groups (p<0.05-p<0.01; Figure 5D).

In the basal zone, the increase was detected at the gene level only at 19 dg and 21 dg compared to other groups (p<0.001; Figure 5B). The Bcl2 gene expression increased in the basal zone with the progression of gestation from 16 dg to 19 dg and 21 dg (p<0.001, Figure 5B, Appendix C). Both the Bcl2 gene and Bcl2 protein expressions were recovered in the progesterone-restored groups in the labyrinth and basal zones (p<0.05-p<0.001; Figures 5B, 5D).

**Figure 6:**
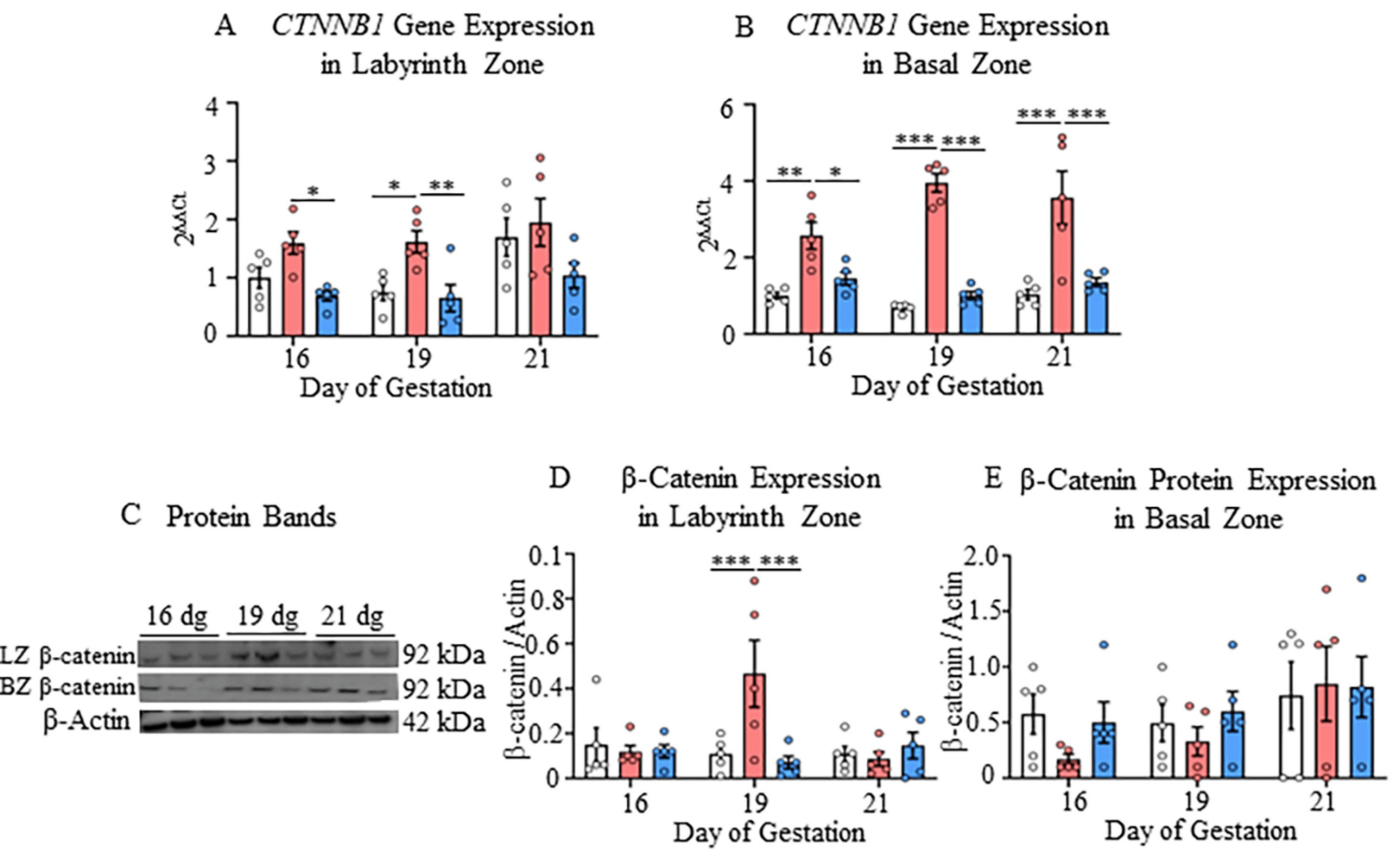
Gene and protein expressions of β-catenin in the labyrinth and basal zones A) Gene expression of CTNNB1 in the labyrinth and B) Basal zone; C) Protein bands of β-catenin and β-actin in the nuclear fraction of labyrinth and basal zones; D) Protein expression of β-catenin in the labyrinth and E) Basal zones at 16, 19, and 21 dg in control, progesterone-reduced, and restored-progesterone groups. ^*^Represents significant differences between groups at the same gestational age using two-way ANOVA (^*^p<0.05, ^**^p<0.01, ^***^p<0.001). Data are represented as mean ± SEM (n=5)

The β-catenin gene expression also increased significantly in both placental zones in the progesterone-reduced group compared to other groups at all gestational ages. In the labyrinth zone, the increased expression of β-catenin was seen at gene and protein levels at 19 dg compared to other groups (p<0.05-p<0.001; Figures 6A, 6D).

**Figure 7:**
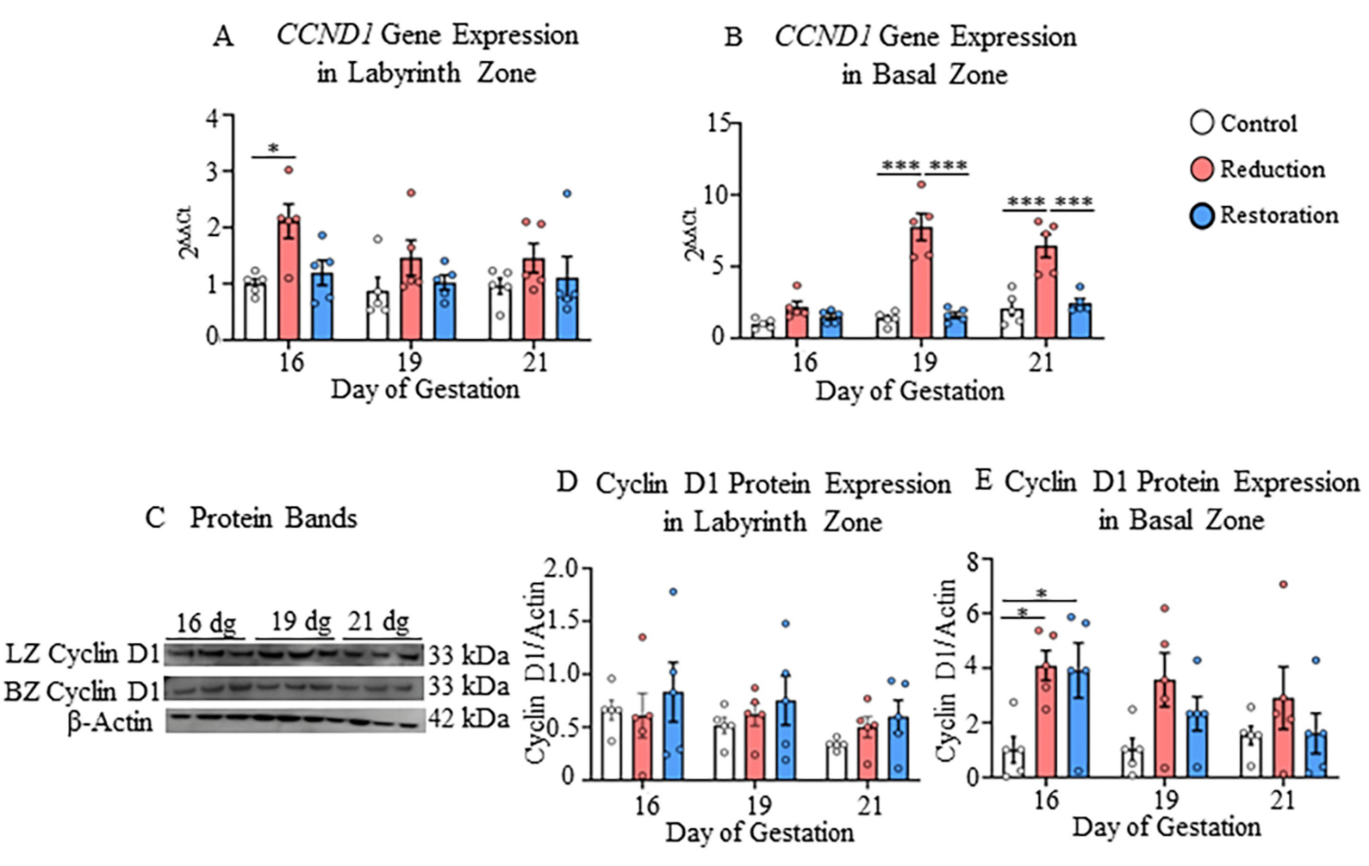
Gene and protein expressions of cyclin D1 in labyrinth and basal zones A) Gene expression of CCND1 in labyrinth and B) Basal zones; C) Protein bands of cyclin D1 and β-actin in the nuclear fraction of labyrinth and basal zones; D) Protein expression of cyclin D1 in labyrinth and E) Basal zones at 16, 19, and 21 dg in control, progesterone-reduced, and restored-progesterone groups. ^*^Represents significant differences between groups at the same gestational age; ^#^represents significant differences between similar groups at different gestational ages using two-way ANOVA (^**^p<0.001). Data are represented as mean ± SEM (n=5)

In the basal zone, however, the higher expression of the CTNNB1 gene was detected at all gestational ages (p<0.05-p<0.001; Figure 6B). The CTNNB1 gene expression in the progesterone-reduced group increased in the basal zone with the progression of gestation (p<0.05, Appendix D). Changes in the level of cyclin D1 were detected in the basal zone only at the gene level. The CCND1 gene expression showed a significant increase at 19 dg and 21 dg compared to the control and progesterone-restored groups (p<0.001; Figure 7B).

The progesterone level restoration recovered the gene and protein expressions of CTNNB1 and CCND1 in both the placental zones (Figures 6A, 6B, 6D, 7B).

## Discussion

Significance of progesterone levels in the induction of growth retardation in rats

Generally, during pregnancy, maternal progesterone levels increase and then decline towards the end of gestation [28]. This is believed to be due to higher apoptotic activity in the basal zone and a reduced demand for high levels of sex steroids during late pregnancy (reviewed in [20]). This trend was also shown in our study, suggesting that the experimental protocol was in line with the accepted progesterone level variations in pregnancy, and the variation in the hormone profile correlated with increasing fetal body weight from 16 dg to 21 dg. When the data from progesterone levels, fetal and placental weights were considered together, a 37% decrease in progesterone level on 19 dg resulted in a reduction in placental weight due to reduced basal zone weight, suggesting to the fact that placental steroidogenesis was affected. That said, the lack of effects on the labyrinth zone indicates that the nutritional supply to the developing fetus was unaffected. In fact, ovariectomy on 15 dg induced an immediate effect on the basal zone as reflected by its weight reduction on 16 dg, which extended to 19 dg, but without affecting the placenta on 21 dg, suggesting that regardless of the progesterone levels, fetal and placental weights are maintained at full term. 

Significance of partial progesterone deprivation-mediated changes in apoptosis- and cell survival- related genes and proteins in the labyrinth zone

The labyrinth zone showed upregulation of the Tp53 gene on 16 dg and 19 dg and its inducible genes CDKN1A and CDKN1B, and a pro-apoptotic Bax gene and protein on 16 dg in progesterone-reduced rats, suggesting cell cycle arrest and apoptosis [29]. Bax is a pro-apoptotic protein that promotes mitochondrial membrane permeabilization and cell death, and its upregulation suggests enhanced apoptotic signaling in response to progesterone deprivation. However, concomitantly, Bcl2 protein (upregulated on 21 dg), cyclin D1 gene (CCND1; upregulated on 16 dg), and β-catenin gene (CTNNB1) and protein (upregulated on 19 dg) also showed upregulation, indicating a co-existing cell survival mechanism, which may be the reason for the lack of reduction in the labyrinth zone weight. This may also be related to a compensatory mechanism designed to maintain a constant cell number, or it may be because of placental zone-specific and cell cycle-specific regulatory mechanisms. As such, the labyrinth zone cells proliferate at a higher rate than the basal zone, which might have contributed to the unchanged labyrinth zone weight.

Partial progesterone withdrawal results in the upregulation of pro-apoptotic proteins and a reduction in the weight of the basal zone

In contrast to the labyrinth zone, the basal zone showed a reduction in its weight. Progesterone deprivation increased p53 gene (Tp53) on 16 dg and 19 dg, which increased p21 gene (CDKN1A) on 21 dg and p27 gene (CDKN1B) on 19 dg and 21 dg, along with upregulation of Bax gene (Bax) and protein, suggesting cell death in the basal zone, which may be the reason for the reduction in its weight. The cell survival mechanism in the basal zone was not efficient, as only pro-cell survival genes- Bcl2 (BCL2) and β-catenin (CTNNB1) increased, and not their proteins. Moreover, the basal zone is also a hormone-dependent region of the placenta, and as there was a significant reduction in progesterone levels, the basal zone weight was decreased. 

The β-catenin protein is activated in the Wnt signaling pathway, and it induces the expression of cyclin D1. In the present study, the increased gene level of cyclin D1 (CCND1) was correlated with the higher gene expression of β-catenin (CTNNB1) detected in the placental basal zone. It could also be increased as a direct effect of higher apoptotic activity and the upregulation of p53 expression [11]. Both p21 and p27 are known to cause cyclin D1 translocation to the nucleus, which has a dual role in inducing apoptosis and proliferation [30]. The higher expression of cyclin D1 gene (CCND1) could be an attempt to increase proliferation and maintenance of the basal zone size, or it could be involved in apoptosis. The increase in anti-apoptotic markers (β-catenin (CTNNB1) and Bcl2 (BCL2) genes) in the basal zone was seen only at the gene level. This suggests that the increased apoptotic activity seen in the basal zone was not opposed by anti-apoptotic genes, resulting in increased apoptosis and a smaller size of the basal zone, and possibly decreasing the steroidogenic function of this zone.

Progesterone imparts zone-specific placental growth effects in rats

Low progesterone level is known to induce apoptosis in uterine and trophoblast cells [15,18], and progesterone supplementation decreases the expression of pro-apoptotic proteins and increases the expression of anti-apoptotic proteins in different cells and tissues [16,17]. In the present study, the progesterone withdrawal effect was translated as a reduction in placental zone weights, which is known to be tissue-specific, as seen in the reproductive organs [26]. The selective zone-specific progesterone effects in placentas showed a significant reduction in the weight of the basal zone and no change in the weight of the labyrinth zone. This effect on the basal zone could be due to higher expression of progesterone receptors, leading to increased sensitivity compared to the labyrinth zone [2]. The increased expression of progesterone receptors in the basal zone would show a greater response to lower maternal progesterone levels, which was translated as a significant reduction in the weight of this zone. The selective effect of progesterone deprivation on the different placental zones could also be due to the labyrinth zone's higher proliferative nature, which compensated for the higher expression of pro-apoptotic genes in both placental zones. Anti-apoptotic proteins also showed a significant upregulation in the progesterone-reduced group, possibly as a compensatory mechanism to preserve placental weight, opposing the high apoptotic activity. These compensatory activities appear to restore the labyrinth zone weight and maintain its vital function in safeguarding fetal body weight and growth, but not in the basal zone.

A major limitation of the study is that the control group did not undergo mini-pump implantation. This was primarily due to the limited availability of mini-pumps and time constraints that prevented the extension of the sample collection period. The other limitation involved the use of only one progesterone-reduced group, in which IUGR did not develop. Including an additional group with a lower level of progesterone support could have helped clarify the relationship between reduced progesterone and the development of IUGR, thereby strengthening the results.

## Conclusions

The findings of the present study indicate that progesterone has a clear anti-apoptotic effect in the labyrinth zone, as the size of the zone was maintained. However, the anti-apoptotic effect in the basal zone was less effective as the weight of the basal zone decreased. These findings clearly emphasize that the effect of progesterone is zone-specific. It also outlines the role of progesterone in apoptosis-proliferation balance and how it is important for the well-being of the placenta and the fetus. 

The zone-specific effect of progesterone seen in the present study highlights the need for further investigation into molecular mechanisms underlying differential placental responses, including signaling pathways, receptor profile in the labyrinth and basal zone.

## Appendices

Appendix A 

**Table 4:** The significant difference in gross data between corresponding groups at different gestational ages The significant difference value between 16 vs. 19, 16 vs. 21 and 19 vs. 21 in progesterone level, fetal body weight, placental weight, labyrinth zone weight, basal zone weight and placental efficiency using two-way ANOVA.

Parameter	Comparison groups	Significant		
		Control	Progesterone-reduced	Progesterone-restored
Progesterone Level	16 vs. 19	0.004	ns	ns
	16 vs. 21	ns	ns	ns
	19 vs. 21	<0.001	ns	ns
Fetal Body Weight	16 vs. 19	<0.001	<0.001	<0.001
	16 vs. 21	<0.001	<0.001	<0.001
	19 vs. 21	<0.001	<0.001	<0.001
Placental Weight	16 vs. 19	<0.001	0.003	0.005
	16 vs. 21	<0.001	<0.001	0.003
	19 vs. 21	ns	ns	ns
Labyrinth Zone Weight	16 vs. 19	<0.001	<0.001	<0.001
	16 vs. 21	<0.001	<0.001	<0.001
	19 vs. 21	ns	ns	ns
Basal Zone Weight	16 vs. 19	ns	ns	ns
	16 vs. 21	0.007	ns	ns
	19 vs. 21	ns	ns	ns
Placental Efficiency	16 vs. 19	<0.001	<0.001	<0.001
	16 vs. 21	<0.001	<0.001	<0.001
	19 vs. 21	<0.001	<0.001	<0.001

Appendix B 

**Table 5:** The significant difference in gene and protein expressions of p53 and gene expression of CDKN1A and CDKN1B in labyrinth and basal zones between corresponding groups at different gestational ages The significant difference value between 16 vs. 19, 16 vs. 21 and 19 vs. 21 using two-way ANOVA.

Parameter	Comparison groups	Significant		
		Control	Progesterone-reduced	Progesterone-restored
Tp53 Gene Expression in Labyrinth Zone	16 vs. 19	ns	ns	ns
	16 vs. 21	ns	ns	ns
	19 vs. 21	0.035	ns	ns
Tp53 Gene Expression in Basal Zone	16 vs. 19	ns	0.003	ns
	16 vs. 21	ns	<0.001	ns
	19 vs. 21	0.035	ns	ns
p53 Protein Expression in Labyrinth Zone	16 vs. 19	ns	ns	ns
	16 vs. 21	ns	ns	ns
	19 vs. 21	ns	ns	ns
p53 Protein Expression in Basal Zone	16 vs. 19	ns	ns	ns
	16 vs. 21	ns	ns	ns
	19 vs. 21	ns	ns	ns
CDKN1A Gene Expression in Labyrinth Zone	16 vs. 19	ns	0.001	ns
	16 vs. 21	0.001	<0.001	0.003
	19 vs. 21	0.003	0.019	0.008
CDKN1A Gene Expression in Basal Zone	16 vs. 19	0.048	ns	0.005
	16 vs. 21	ns	0.004	ns
	19 vs. 21	0.037	<0.001	0.020
CDKN1B Gene Expression in Labyrinth Zone	16 vs. 19	ns	<0.001	<0.001
	16 vs. 21	<0.001	0.007	0.003
	19 vs. 21	0.003	0.019	0.008
CDKN1B Gene Expression in Basal Zone	16 vs. 19	ns	ns	0.005
	16 vs. 21	ns	0.004	ns
	19 vs. 21	0.037	<0.001	0.020

Appendix C 

**Table 6:** The significant difference in gene and protein expressions of Bax and Bcl2 in labyrinth and basal zones between corresponding groups at different gestational ages The significant difference value between 16 vs. 19, 16 vs. 21 and 19 vs. 21 using two-way ANOVA.

Parameter	Comparison groups	Significant		
		Control	Progesterone-reduced	Progesterone-restored
Bax Gene Expression in Labyrinth Zone	16 vs. 19	ns	ns	ns
	16 vs. 21	ns	ns	ns
	19 vs. 21	0.035	ns	0.038
Bax Gene Expression in Basal Zone	16 vs. 19	0.021	ns	0.014
	16 vs. 21	ns	0.009	ns
	19 vs. 21	ns	0.049	ns
Bax Protein Expression in Labyrinth Zone	16 vs. 19	ns	ns	ns
	16 vs. 21	ns	ns	ns
	19 vs. 21	ns	ns	ns
Bax Protein Expression in Basal Zone	16 vs. 19	ns	ns	ns
	16 vs. 21	0.019	0.002	ns
	19 vs. 21	0.027	0.026	ns
Bcl2 Gene Expression in Labyrinth Zone	16 vs. 19	ns	ns	ns
	16 vs. 21	ns	ns	ns
	19 vs. 21	ns	ns	ns
Bcl2 Gene Expression in Basal Zone	16 vs. 19	ns	<0.001	ns
	16 vs. 21	ns	<0.001	ns
	19 vs. 21	ns	ns	ns
Bcl2 Protein Expression in Labyrinth Zone	16 vs. 19	ns	ns	ns
	16 vs. 21	ns	ns	ns
	19 vs. 21	ns	ns	ns
Bcl2 Protein Expression in Basal Zone	16 vs. 19	ns	ns	ns
	16 vs. 21	ns	ns	ns
	19 vs. 21	ns	ns	ns

Appendix D 

**Table 7:** The significant difference in gene and protein expressions of β-catenin and cyclin D1 in labyrinth and basal zones between corresponding groups at different gestational ages The significant difference value between 16 vs. 19, 16 vs. 21 and 19 vs. 21 using two-way ANOVA.

Parameter	Comparison groups	Significant		
		Control	Progesterone-reduced	Progesterone-restored
CCTNB1 Gene Expression in Labyrinth Zone	16 vs. 19	ns	ns	ns
	16 vs. 21	ns	ns	ns
	19 vs. 21	0.007	ns	ns
CCTNB1 Gene Expression in Basal Zone	16 vs. 19	ns	ns	ns
	16 vs. 21	ns	ns	ns
	19 vs. 21	ns	ns	ns
β-catenin Protein Expression in Labyrinth Zone	16 vs. 19	ns	<0.001	ns
	16 vs. 21	ns	ns	ns
	19 vs. 21	ns	<0.001	ns
β-catenin Protein Expression in Basal Zone	16 vs. 19	ns	ns	ns
	16 vs. 21	ns	ns	ns
	19 vs. 21	ns	ns	ns
CCND1 Gene Expression in Labyrinth Zone	16 vs. 19	ns	ns	ns
	16 vs. 21	ns	ns	ns
	19 vs. 21	ns	ns	ns
CCND1 Gene Expression in Basal Zone	16 vs. 19	ns	0.026	ns
	16 vs. 21	ns	0.042	ns
	19 vs. 21	ns	ns	ns
Cyclin D1 Protein Expression in Labyrinth Zone	16 vs. 19	ns	ns	ns
	16 vs. 21	ns	ns	ns
	19 vs. 21	ns	ns	ns
Cyclin D1 Protein Expression in Basal Zone	16 vs. 19	ns	ns	ns
	16 vs. 21	ns	ns	ns
	19 vs. 21	ns	ns	ns

## References

[1] Diemert A, Goletzke J, Barkmann C, Jung R, Hecher K, Arck P (2017). Maternal progesterone levels are modulated by maternal BMI and predict birth weight sex-specifically in human pregnancies. J Reprod Immunol.

[2] Mark PJ, Smith JT, Waddell BJ (2006). Placental and fetal growth retardation following partial progesterone withdrawal in rat pregnancy. Placenta.

[3] Alawadhi M, Mouihate A, Kilarkaje N, Al-Bader M (2022). Progesterone partially recovers placental glucose transporters in dexamethasone-induced intrauterine growth restriction. Reprod Biomed Online.

[4] Alqaryyan M, Kilarkaje N, Mouihate A, Al-Bader MD (2017). Dexamethasone-induced intrauterine growth restriction is associated with altered expressions of metastasis tumor antigens and cell cycle control proteins in rat placentas. Reprod Sci.

[5] Alawadhi MM, Al Shammari F, Ali FM, Almatar R, Al-Duwaikhi A, Al-Bader MD (2022). The effect of progesterone administration on the expression of metastasis tumor antigens (MTA1 and MTA3) in placentas of normal and dexamethasone-treated rats. Mol Biol Rep.

[6] Hernández Borrero LJ, El-Deiry WS (2021). Tumor suppressor p53: biology, signaling pathways, and therapeutic targeting. Biochim Biophys Acta Rev Cancer.

[7] Ma Q, Yu J, Zhang X, Wu X, Deng G (2023). Wnt/β-catenin signaling pathway-a versatile player in apoptosis and autophagy. Biochimie.

[8] Kikuchi A (2000). Regulation of beta-catenin signaling in the Wnt pathway. Biochem Biophys Res Commun.

[9] Yao G, Tang J, Yang X (2020). Cyclin K interacts with β-catenin to induce Cyclin D1 expression and facilitates tumorigenesis and radioresistance in lung cancer. Theranostics.

[10] Engeland K (2022). Cell cycle regulation: p53-p21-RB signaling. Cell Death Differ.

[11] Damalas A, Kahan S, Shtutman M, Ben-Ze'ev A, Oren M (2001). Deregulated beta-catenin induces a p53- and ARF-dependent growth arrest and cooperates with Ras in transformation. EMBO J.

[12] Kakkar V, Sarin V, Chatterjee A, Manjari M, Chopra I (2022). Expression of cyclin-D1 and p53 as prognostic markers in treatment of oral squamous cell carcinoma. Indian J Otolaryngol Head Neck Surg.

[13] Rocha S, Martin AM, Meek DW, Perkins ND (2003). p53 represses cyclin D1 transcription through down regulation of Bcl-3 and inducing increased association of the p52 NF-kappaB subunit with histone deacetylase 1. Mol Cell Biol.

[14] Rider V, Isuzugawa K, Twarog M, Jones S, Cameron B, Imakawa K, Fang J (2006). Progesterone initiates Wnt-beta-catenin signaling but estradiol is required for nuclear activation and synchronous proliferation of rat uterine stromal cells. J Endocrinol.

[15] Liu J, Matsuo H, Laoag-Fernandez JB, Xu Q, Maruo T (2007). The effects of progesterone on apoptosis in the human trophoblast-derived HTR-8/SV neo cells. Mol Hum Reprod.

[16] Wang Y, Abrahams VM, Luo G, Norwitz NG, Snegovskikh VV, Ng SW, Norwitz ER (2018). Progesterone inhibits apoptosis in fetal membranes by altering expression of both pro- and antiapoptotic proteins. Reprod Sci.

[17] Yin P, Lin Z, Cheng YH (2007). Progesterone receptor regulates Bcl-2 gene expression through direct binding to its promoter region in uterine leiomyoma cells. J Clin Endocrinol Metab.

[18] Morimoto H, Ueno M, Tanabe H, Kono T, Ogawa H (2021). Progesterone depletion results in Lamin B1 loss and induction of cell death in mouse trophoblast giant cells. PLoS One.

[19] Tian F, Han H, Jia L (2022). The effects of mifepristone on the structure of human decidua and chorion and Bax and Bcl-2 expression at early stage of pregnancy. BMC Pharmacol Toxicol.

[20] Furukawa S, Hayashi S, Usuda K, Abe M, Hagio S, Ogawa I (2011). Toxicological pathology in the rat placenta. J Toxicol Pathol.

[21] Xie XM, Cao QL, Sun YJ (2023). LRP6 bidirectionally regulates insulin sensitivity through insulin receptor and S6K signaling in rats with CG-IUGR. Curr Med Sci.

[22] Menendez-Castro C, Toka O, Fahlbusch F (2014). Impaired myocardial performance in a normotensive rat model of intrauterine growth restriction. Pediatr Res.

[23] Song J, Cho J, Park J, Hwang JH (2022). Identification and validation of stable reference genes for quantitative real time PCR in different minipig tissues at developmental stages. BMC Genomics.

[24] Al-Bader MD, Kilarkaje N, El-Farra A, Al-Abdallah AA (2015). Expression and subcellular localization of metastasis-associated protein 1, its short form, and estrogen receptors in rat placenta. Reprod Sci.

[25] Falahzadeh K, Banaei-Esfahani A, Shahhoseini M (2015). The potential roles of actin in the nucleus. Cell J.

[26] Alawadhi M, Kilarkaje N, Mouihate A, Al-Bader MD (2023). Role of progesterone on dexamethasone-induced alterations in placental vascularization and progesterone receptors in rats. Biol Reprod.

[27] Schneider CA, Rasband WS, Eliceiri KW (2012). NIH image to ImageJ: 25 years of image analysis. Nat Methods.

[28] Hashimoto H, Eto T, Endo K, Itai G, Kamisako T, Suemizu H, Ito M (2010). Comparative study of doses of exogenous progesterone administration needed to delay parturition in Jcl:MCH(ICR) mice. Exp Anim.

[29] Fragkos M, Jurvansuu J, Beard P (2009). H2AX is required for cell cycle arrest via the p53/p21 pathway. Mol Cell Biol.

[30] Cheng M, Olivier P, Diehl JA, Fero M, Roussel MF, Roberts JM, Sherr CJ (1999). The p21(Cip1) and p27(Kip1) CDK 'inhibitors' are essential activators of cyclin D-dependent kinases in murine fibroblasts. EMBO J.

